# Evaluation of the impact of levothyroxine treatment on the psychomotor developmental status of three-year-old children born to mothers with mild thyroid impairment; Tehran Thyroid and pregnancy study: study protocol for a randomized clinical trial

**DOI:** 10.1186/s13063-018-3130-5

**Published:** 2019-01-28

**Authors:** Sima Nazarpour, Fahimeh Ramezani Tehrani, Firoozeh Sajedi, Razieh Bidhendi Yarandi, Fereidoun Azizi

**Affiliations:** 1 Department of Midwifery, Varamin-Pishva Branch, Islamic Azad University, Tehran, Iran; 2 Reproductive Endocrinology Research Center, Research Institute for Endocrine Sciences, Shahid Beheshti University of Medical Sciences, Tehran, Iran; 3 Pediatric Neurorehabilitation Research Center, University of Social Welfare & Rehabilitation Sciences, Tehran, Iran; 4 Department of Epidemiology and Biostatistics, School of Public Health, Tehran University of Medical Sciences, Tehran, Iran; 5 Endocrine Research Center, Research Institute for Endocrine Sciences, Shahid Beheshti University of Medical Sciences, Tehran, Iran

**Keywords:** Neurodevelopmental, Offspring, Protocol, Subclinical hypothyroidism, Pregnancy, Clinical trial

## Abstract

**Background:**

Despite the known adverse effects of maternal overt hypothyroidism on the neurocognitive development of children, there is uncertainty regarding the impact of gestational thyroid dysfunction or autoimmune thyroiditis on infant/child neurological development. This study aims to evaluate the impact of levothyroxine (LT4) treatment on the psychomotor developmental status of three-year-old children born to mothers with mild thyroid impairment (subclinical hypothyroidismwith/without autoimmune thyroiditis).

**Methods/Design:**

This is a follow-up study of the Tehran Thyroid and Pregnancy Study, a randomized trial in which subclinical hypothyroid pregnant women were assigned to an intervention group (treated with levothyroxine) or a control group (received no treatment). The primary outcome for the purpose of the present study is the developmental status of the children, aged three years, in five domains (communication, gross motor, fine motor, problem-solving, and social–personal domains) using the Ages and Stages Questionnaire (ASQ).

**Discussion:**

The study is designed to assess the developmental status of children born to mothers with mild thyroid impairment (subclinical hypothyroidism with/without autoimmune thyroiditis). This study is one of the limited studies available in this field and has the potential to facilitate much-needed information for related public health policies.

**Trial registration:**

Iranian Registry of Clinical Trials, IRCT2017090314849N5. Registered on 11 September 2017. Iranian Registry of Clinical Trials, IRCT2017090414849N6. Registered on 14 October 2017.

**Electronic supplementary material:**

The online version of this article (10.1186/s13063-018-3130-5) contains supplementary material, which is available to authorized users.

## Background

Several studies revealed the distinguishable role of maternal thyroid hormones on all stages of brain cell development [1, 2]. In the first trimester of pregnancy, a time when the embryo’s thyroid is still developing, fetal thyroid hormones are completely dependent on the mother as a source. However, after this stage, the mother and fetus both contribute to this need. Studies revealed the presence of specific nuclear receptors and thyroid hormones in the fetal brain at the eight week of gestation and free T4 in the coelomic and amniotic fluids, underling the role of thyroid hormones in early fetal brain development [3].

Both overt hypothyroidism and hyperthyroidism are well-known modifiable risk factors for various fetomaternal and child outcomes [4, 5]. Data regarding the adverse pregnancy and child outcomes of subclinical forms of thyroid dysfunction or thyroid autoimunity are inconclusive and treatment benefits in such women are unclear. Despite observational studies reporting significant association between mild gestational thyroid dysfunctions and several pregnancy complications or neurocognitive impairment in the offspring [6–14], treatment of subclinical hypothyroidism (SCH) or hypothyroxenemia have not improved pregnancy outcomes or even cognitive functions in their children in limited randomized clinical trials (RCT) carried out among pregnant women [15–17]. The results of studies on the effects of maternal autoimmune thyroid disorders on cognitive development in children also differ. Pop et al. in 1995 reported that the neurodevelopmental scores of children of TPOAb-positive (TPOAb^+^) mothers were significantly worse than those with TPOAb-negative (TPOAb^−^) mothers [18]; in contrast, Williams et al. [19] demonstrated no evidence of an association between maternal TPOAb and TSH levels and neurodevelopmental outcome. Li et al. [11] showed that the intellectual and motor development of children aged 25–30 months is associated with abnormalities of maternal thyroid at 16–20 weeks’ gestation; maternal SCH or euthyroidism with elevated TPOAb titers were found to be predictors of lower motor and intellectual development. Ghassabian et al. [20] showed that higher titers of TPOAbs during pregnancy were associated with an increase in children’s risk of attention deficit/hyperactivity; however, this adverse effect was not observed in the Controlled Antenatal Thyroid Screening (CATS) study [21, 22] that investigated treatment for SCH on childhood cognition and found no differences in IQ at the ages of three years and 9.5 years between children of treated and untreated SCH mothers. In addition, studies by Casey et al. (2017) and Lazarus et al. (2012) showed maternal treatment for SCH did not result in improved cognitive function in children [15, 17].

Hence, considering the fact that the effect of treatment on the development status of offspring is not clear, in this follow-up clinical trial study, we aim to evaluate the impact of levothyroxine (LT4) treatment on the psychomotor developmental status of three-year-old children born to mothers with mild thyroid impairment (SCH with/without autoimmune thyroiditis).

## Methods

### Overall trial design

This is the follow-up study from The Tehran Thyroid and Pregnancy Study [23], a two-phase population-based RCT carried out on pregnant women referring to prenatal clinics of Shahid Beheshti University of Medical Sciences. In brief, in the first phase of that study, using a cluster-sampling method, first-trimester pregnant women were screened for thyroid disorders; in the second phase of the study, those with SCH (both TPOAb-positive and -negative) were randomly assigned to be treated with levothyroxine (intervention) or to receive no treatment. The details of the original study have been published before [23].

The Tehran Thyroid and Pregnancy Study was conducted from September 2013 through February 2016. The first phase of this study was a population-based cross-sectional study in which 1746 pregnant women attending the prenatal clinics of Shahid Beheshti University of Medical Sciences were screened for thyroid dysfunction. Overnight blood samples were collected at the first prenatal visit and second (20–24 weeks of gestation) and third (30–34 weeks of gestation) trimesters to measure serum levels of TSH, thyroxine (T4), resin T-uptake (RTU), and TPOAb. Table 1 shows the classification of mothers according to the level of TSH, FT4I, and TPOAb.

**Table 1:** Classification of mothers according to the level of TSH, FT4I, and TPOAb

Classification	TSH (mIU/L)	FT4I	TPOAb (IU/mL)
Euthyroid	0.1–2.5	1–4.5	< 50
Overt hyperthyroidism	< 0.1	> 4.5	–
SCH	< 0.1	1–4.5	–
Overt hypothyroidism	> 10	-	–
Overt hypothyroidism	> 2.5	< 1	–
SCH TPOAb^−^	2.5–10	1–4.5	< 50
SCH TPOAb^+^	2.5–10	1–4.5	> 50

*TPOAb* thyroid peroxidase antibodies, *SCH* subclinical hypothyroidism

At the first prenatal visit, participants were asked to collect three casual morning urine samples (5–10 mL) every other day.

**Fig. 1:**
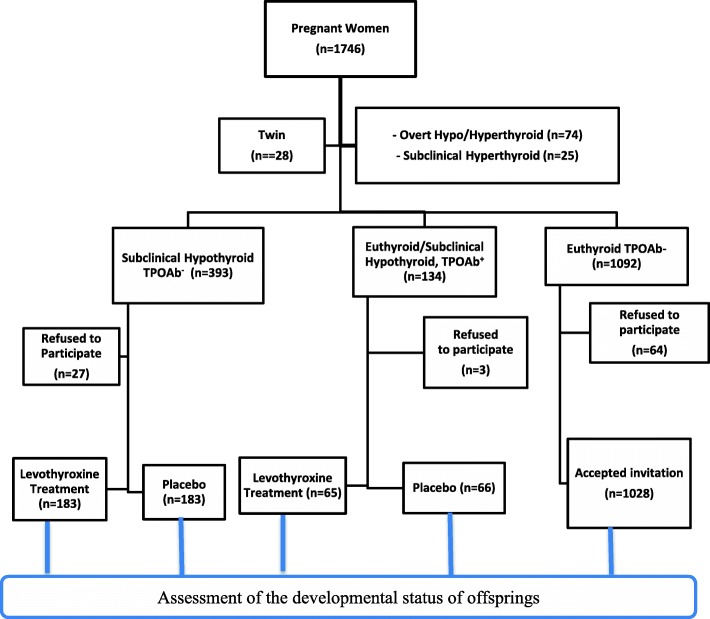
Study *flow chart*

After excluding those with twin pregnancies and those with overt thyroid dysfunction or SCH (*n* = 127), euthyroid women and those with other types of thyroid dysfunction (*n* = 1619) were invited for the second phase of the study. Two RCTs, one of TPOAb^+^ cases (*n* = 131) [24] and the other of SCH–TPOAb^−^ women (*n* = 366) [25], were conducted in the second phase of the study; the remaining 1028 euthyroid TPOAb^−^ women served as controls and were followed until delivery (Fig. 1).

SCH–TPOAb^−^ women and TPOAb^+^ cases were randomly assigned into two groups (using a computer-generated list) to achieve balance across treatment groups, according to permuted blocks of four; the LT4 treatment group was treated with LT4 and the placebo group went without treatment. Women in the LT4 treatment group received LT4: a morning dose of 1 μg/kg/day, a dose maintained throughout the pregnancy. In brief, for the purpose of the present study, we have five groups including two cases (TPOAb^+^ treated with LT4 and SCH–TPOAb^−^ treated with LT4) and three controls (TPOAb^+^ not treated with LT4, SCH–TPOAb^−^ not treated with LT4, and euthyroid TPOAb^−^ women).

### Neurodevelopmental study of offsprings

Both groups of children (aged three years) born to mothers with SCH and/or autoimmune thyroid disease will be invited to participate. In the present study, parents will complete the Ages and Stages Questionnaire to assess the children’s developmental status. The children of those euthyroid TPOAb^−^ women will also be invited to be participate in this follow-up study and serve as controls (Fig. 1).

Participants will be contacted by phone; the aim and purpose of the present study will be explained in detail. An appointment will be arranged for those participants who wish to complete the development assessment. A trained examiner will explain the instructions for completing the questionnaire. All assessments will be performed by one examiner only to maximize reliability. A randomly selected 5% of the completed assessments will be retested in two weeks and double-scored to ensure the reliability; the scores are calculated and a report is generated for the participants to be taken to their parents. If participants need to be given more information about the results or have any questions, they can contact the examiner directly.

In the present study, the ASQ, a parent-report questionnaire, was chosen because it has been proven to be a valid and/or reliable screening test for Iranian infants and children [26]. The ASQ questionnaire assesses the developmental status of children aged 4–60 months (in 19 age groups) in five developmental domains including communication, gross motor skills, fine motor skills, problem-solving, and social–personal domains. It includes six open-ended questions that assess the parents’ concerns such as visual and auditory problems. Each age-specific domain includes six items, scores are in the range of 0–60 in each subscale, and the total score is in the range of 0–300 [27]. The scoring system is based on a 10–5–0 scoring scale (10 points for every “yes,” 5 points for every “sometimes,” and 0 points for every “not yet” answer to the questions). The sum of scores is then compared with a list of age-specific cut-off points standardized for Iranian children [26, 28].

ASQ validity has been examined across different cultures and communities across the world [29–32] and has excellent psychometric properties (test–retest reliability of 92%, sensitivity of 87.4%, and specificity of 95.7%) [26, 29, 31–33]. The inter-rater reliability values obtained were 0.88, 0.91, 0.88, 0.89, and 0.86 for the communication, gross motor skills, fine motor skills, problem-solving, and social–personal domains, respectively, and 0.93 totally [26]. It had the ability to precisely determine developmental disorders in > 96% of children [34].

**Fig. 2:**
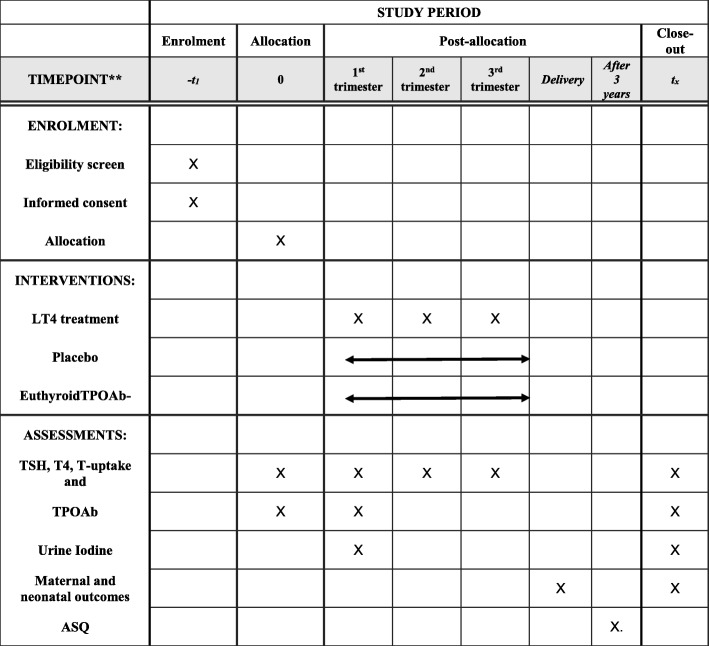
Summary of study events schedule. TPOAb thyroid peroxidase antibodies, TSH thyroid stimulating hormone, LT4 levothyroxine, ASQ Ages and Stages Questionnaires

The study protocol has been designed and reported in line with SPIRIT guidelines, with an attached SPIRIT flow diagram (Fig. 2) and checklist (Additional file 1) [35].

### Ethics approval

Written informed consent will be obtained from all participants by the examiner. It will be emphasized that participation in the study is voluntary and they were free to withdraw from the study at any time.

Both original and current studies have been been approved by the Medical Ethics Committee of the Research Institute for Endocrine Sciences of Shahid Beheshti University of Medical Sciences and received two separate codes for studies among TPOAb^+^ women and among SCH TPOAb^−^ ones (approval code numbers for original study: IR.SBMU.ENDOCRINE.REC.1395.297 and IR.SBMU.RIES.REC.1394.48, approval code numbers for current study IR.SBMU.ENDOCRINE.REC.1395.299 and IR.SBMU.ENDOCRINE.REC.1395.300). They also were registered in the Iranian Randomized Clinical Trials (IRCT) Registry (trial codes for original study: IRCT2013100114849N1, IRCT2013112014849N2, and IRCT2013121214849N3; trial codes for current study: IRCT2017090414849N6 and IRCT2017090314849N5).

### Power calculation and sample size justification

The purpose of this study will be multiple comparisons of five groups: three groups of controls: no intervention TPOAb^+^, no intervention TPOAb^−^, and healthy; as well as two cases: intervention TOPAb^+^ and TPOAb^−^. We will consider five primary outcomes: communication; gross motor skills; fine motor skills; problem-solving; and social–personal aspects.

Post-hoc power calculation was done to detect samples needed. It has been shown that, with power of 80% and type one error of 5%, nearly 2356, 590, 263, 149, 96, 67, 50, 39, and 31 participants are required to find the effect sizes 0.1, 0.2, 0.3, 0.4, 0.5, 0.6, 0.7, 0.8, and 0.9 statistically significant. We will consider q = 15% attrition as well which increases sample size by n* = n/1-q. The following formula for comparison of groups is applied:

$$ \boldsymbol{n}\boldsymbol{\ge}\left(\mathbf{1}+\sqrt{\boldsymbol{g}-\mathbf{1}}\right)\frac{{\left({\boldsymbol{z}}_{\mathbf{1}-\frac{\boldsymbol{\alpha}}{\mathbf{2}}}+{\boldsymbol{z}}_{\mathbf{1}-\boldsymbol{\beta}}\right)}^{\mathbf{2}}}{{\boldsymbol{d}}^{\mathbf{2}}}+\frac{{\boldsymbol{z}}_{\mathbf{1}-\frac{\boldsymbol{\alpha}}{\mathbf{2}}}^{\mathbf{2}}\sqrt{\boldsymbol{g}-\mathbf{1}}}{\mathbf{2}+\left(\mathbf{1}+\sqrt{\boldsymbol{g}-\mathbf{1}}\right)} $$

### Randomization and blinding

The pregnant women who participated in the original Tehran Thyroid and Pregnancy Study, with a diagnosis of SCH and/or autoimmune thyroid diseases, were randomly allocated into two groups using a computer-generated list in blocks of four. For the purpose of the current study, the trained examiner who assesses the developmental status of children born from those pregnant women using the ASQ are blinded to the previous grouping of participants.

### Statistical analysis

Intention to treat (ITT) analysis will be considered to deal with non-compliance and missing outcomes in our RCT.

Dataset accuracy will be checked using histograms and cross-tabulations to identify any outliers and errors. We will also run “Grubbs” test, an ESD (method extreme studentized deviate), to determine whether any values are significant outliers or implausible values > 4 SD from the mean will be considered as outliers and discarded. Descriptive statistics will be presented as means ± SD for normal, medians and IQR for non-normal variables, and categorical variables presented as percent.

Normality assumption will be checked by Kolmogorov–Smirnov and Shapiro–Wilk test; in case of rejection, the Mann–Whitney non-parametric U test will be applied, otherwise independent Student’s t test will be used to find the differences. For categorical variables, chi-squared test and/or Fisher’s exact test in case of a sparse data problem (cell < 5) will be used. Subgroup analysis for important confounding variables will be run, if needed.

The primary analysis for the study is to assess the effect of treatment on the aspects of psychomotor developmental status. Therefore generalized linear models (GLM) will be conducted modeling the variables and checking the causal relationships between the outcome of interests and other covariates such as treatment. Crude and adjusted models for potentially confounding effects will be fitted.

Interaction effect of treatment and some other covariates will be added to the model and a subgroup analysis will be run to check the effect of treatment on the levels of covariates, variables such as parity, socioeconomic status, type of delivery, lactic status, urinary iodine, and so on.

Since our study follows up the second generation of the ancestors whom intake the intervention, the casual relationships could be assessed via transitional models. There is a series of statistical analyses to estimate these relationships; we use transitional models which applied Markov Chains stochastic process and multistate models.

Statistical analysis will be performed using STATA software (version 10; STATA, Inc., College Station, TX, USA) and the latest version of R CRAN, the “msm” package, to analyze multi-state modelling with R; *p* < 0.05 is considered statistically significant.

## Results

Baseline characteristics of neonates according to their mothers’ thyroid status are presented in Table 2.

**Table 2:** Baseline characteristics of neonates according to their mothers’ thyroid status

Characteristics	SCH-TPOAb^−^		SCH-TPOAb^+^		
	LT4 treatment (*n* = 183)	Placebo (*n* = 183)	LT4 treatment (*n* = 65)	Placebo (*n* = 66)	Euthyroid TPOAb^−^ (*n* = 1028)
Gestational age, mean (SD)	38.03 (1.4)	37.9 (1.5)	39.3 (1.3)	38.4 (1.7)	39.4 (1.4)
Birth weight, mean (SD)	3190.82 (455.13)	3203.1 (497.1)	3139.1 (287.6)	3127.7 (523.5)	3236.6 (448.8)
Birth height, mean (SD)	50.1 (2.3)	50.2 (2.7)	49.5 (1.7)	50.3 (1.5)	50.1 (2.3)
Birth head circumference, mean (SD)	34.6 (1.4)	34.7 (1.6)	34.5(1.1)	34.9 (1.4)	34.7 (2.3)
TSH (μIU/mL), Median (IQ)	1.1 (0.5–2.1)	1.1 (0.5–1.9)	1.3 (0.45–1.9)	1.0 (.43–1.9)	0.90 (0.4–1.7)

*SCH* subclinical hypothyroidism, *TPOAb* thyroid peroxidase antibodies, *TSH* thyroid-stimulating hormone, *LT4* levothyroxine

## Discussion

### Principal findings

SCH has been associated with several obstetric complications [36–38], but there is no direct evidence that levothyroxine therapy reduces these risks, especially in those without thyroid autoimmunity or those been identified using the cut-off value of 2.5 μIU/mL. Conflicting results have been reported regarding the association between subclinical thyroid dysfunctions during pregnancy and neurodevelopmental disorders in their offspring [7, 8, 11, 12, 14, 39, 40]. There is currently no evidence that levothyroxine treatment of pregnant women with SCH, especially when initiated after seven weeks of gestation, improves neurocognitive functions in offspring [15–17]. As a result, neither association has recommended universal screening for SCH or thyroid autoimmunity during pregnancy, considering the lack of robust evidence for the benefit from screening and treating mild thyroid insufficiency [41, 42].

There are limited RCTs with sufficient sample sizes that have assessed the beneficiary effect of administration of levothyroxine in pregnant women with SCH in terms of neurocognitive developments [17, 43, 44]. These studies mainly suffer from lack of initiation of treatment in early gestation (before the fetal thyroid gland become functional), do not consider the influencing effect of iodine deficiency (the association between mild thyroid dysfunction and intellectual disability seen in observational study and may be mediated by iodine defficiency and hence not completely ameliorated by levothyroxine), lack precision of the thyroid tests that have been utilized for categorization of the study participants, and do not report whether it includes those with thyroid autoimmunity.

The key strength of the present study is assessing the neurodevelopmental status of the offspring of both groups of TPOAb^+^ cases and SCH-TPOAb^−^ women along with euthyroid TPOAb^−^ women, considering their iodine sufficiency status during pregnancy. We are not aware of any other studies that have considered all these components simultaneously. The other strength of this study is the ASQ and the number of characteristics assessed. First,the Persian version of the ASQ has appropriate validity and reliability for screening developmental disorders in Iran [26]. It has also been proven to be a valid and/or reliable screening test, i.e. it has translated and culturally adapted versions in several studies in different populations of children [28]. In a multinational trial involving 18 countries in Asia, Africa, Europe, North America, and South America reported in 2007, the sensitivity and specificity were determined to be 88% and 82.5%, respectively [45, 46]. It also uses simple and straightforward language; simple illustrations are provided for many items adding to their clarity of meaning, which makes it a feasible and easy-to-use test [47]. It is inexpensive [47, 48] and takes about 10 min to administer [47]. Administration does not require specialized training. This series of developmental screening questionnaires includes five developmental domains: gross motor skills; fine motor skills; communication; problem-solving; and personal–social domains. The questionnaires cover 19 age groups in the range of 4–60 months [49].

The main challenge of this study will be the rate of attrition; however, its pilot study received a response rate of > 80%. The other challenge of this study is lack of awareness of other factors that may influence the developmental status of the children, e.g. nutritional inadequacies [50], although the randomized allocation of study participants minimizes this effect.

By conducting this study, we hope to better understand the beneficiary impact of treatment of mild forms of thyroid dysfunctions during pregnancy, considering the influential effect of iodine status. This piece of knowledge has the potential for evidence-based decision-making regarding the universal screening for thyroid dysfunction and/or thyroid autoimmunity.

In summary, this is the follow-up study of the Tehran Thyroid and Pregnancy Study; a two-phase population-based RCT that aimed to assess the developmental status of children born to mothers with mild thyroid impairment, in terms of SCH with/without autoimmune thyroiditis.

### Trial status

Protocol numbers: IRCT2017090314849N5 and IRCT2017090414849N6.

Patient recruitment is currently ongoing.

Study start date: September 2017.

Primary completion date: February 2019.

Study completion date: March 2019.

## Additional file

Additional file 1: SPIRIT 2013 Checklist: Recommended items to address in a clinical trial protocol and related documents*. (DOC 121 kb)

## Abbreviations

ASQ: Ages and Stages Questionnaire
CATS: Controlled Antenatal Thyroid Screening
FT4I: Free Thyroxin Index
IQ: Intelligence quotient
LT4: Levothyroxine
SCH: Subclinical hypothyroidism
SD: Standard deviation
TPOAb: Thyroid peroxidase antibodies
TSH: Thyroid-stimulating hormone, thyrotropin, thyrotropic hormone
